# Case report: Congenital mesoblastic nephroma

**DOI:** 10.1016/j.ijscr.2023.108233

**Published:** 2023-04-18

**Authors:** Kristen De Wilde, Jamshed Zuberi

**Affiliations:** a St. George's School of Medicine, 3500 Sunrise Hwy, Great River, NY 11739, United States of America; b Department of General Surgery, St. Joseph's University Medical Center, 703 Main St, Paterson, NJ 07503, United States of America

**Keywords:** Nephroma, Congenital, Renal tumor

## Abstract

**Introduction and importance:**

Congenital mesoblastic nephroma is a common benign renal tumor that mainly affects infants below the age of six months (Daskas et al., 2002). Identifying the pathology type is crucial for determining the appropriate plan of action and predicting the patient's prognosis.

**Case presentation:**

A one-day-old Hispanic neonate was referred for surgical evaluation after detecting a LUQ mass. Ultrasound imaging revealed a heterogenous solid mass that infiltrated the hilum of the left kidney. The patient underwent a left radical nephrectomy, and the pathology results indicated that the mass was consistent with the classic type of congenital mesoblastic nephroma. The patient will be closely monitored by nephrology with frequent abdominal ultrasounds.

**Clinical discussion:**

The case describes a one-day-old female baby with an asymptomatic LUQ abdominal mass, which was diagnosed as mesoblastic nephroma. The baby was born full-term with no significant medical history, and after experiencing hypertensive episodes, she underwent a left radical nephrectomy to remove the tumor. Pathology confirmed mesoblastic nephroma, classic type, and the patient was diagnosed with stage I disease since the tumor was entirely resected with no renal vessel involvement. Follow-up ultrasounds were recommended to monitor for recurrence, and chemotherapy may be considered if recurrence occurs (Pachl et al., 2020). Calcium and renin levels should also be monitored (Bendre et al., 2014).

**Conclusion:**

Although congenital mesoblastic nephroma is typically benign, patients require ongoing monitoring for potential paraneoplastic syndromes. Furthermore, certain types of mesoblastic nephroma can progress to malignancy, necessitating close follow-up during the first few years of life.

## Highlights

• A case of congenital mesoblastic nephroma presenting with hypertensive episodes
• A left upper quadrant mass in a neonate should be further investigated.
• High levels of renin and parathyroid hormone can be indicators of mesoblastic nephromas in neonates.

## 1 Introduction

The work presented here has been reported in line with the SCARE criteria [1].

Congenital mesoblastic nephroma (CMN) is a benign mesenchymal renal tumor, and it is the most common renal tumor in neonates and infants less than six months of age [2]. CMN is the cause of 2–4 % of renal tumors in children [3]. It is still exceedingly rare, occurring in only 8 out of 1 million births [2]. CMN is found to have three different pathology types, including classic CMN, cellular CMN, and mixed variant. Cellular CMN is found to be aggressive and is capable of malignancy, recurrence, and metastasis [4]. On the other hand, patients with classic CMN usually have an excellent overall prognosis [4]. Neonates usually have a palpable left upper quadrant abdominal mass [4], [5]. Furthermore, patients with CMN can have hypercalcemia due to the tumor secretion of parathyroid hormone [5]. In 80 % of CMN cases, hypertension is noted due to hyper-reninemia [5]. The purpose of this case report was to discuss how to identify the paraneoplastic syndrome that can arise from CMN and the surgical treatment and follow-up required for these rare cases.

## 2 Case report

The one-day-old Hispanic female baby was referred to the surgical team after an asymptomatic LUQ mass was detected. The baby was born at 39 weeks and two days via repeat C/S (two) to a 39-year-old G5P3 F with negative prenatals. The mother and father denied any alcohol, tobacco, or drug use. The mother, father, and siblings had no known past medical history. Her birth weight was 3.3 kg (7 lb 4 oz), and her length was 49 cm (1 ft 7 in). Apgar at 1 min and 5 min were 8 and 9, respectively.

A prenatal scan revealed that the left kidney appeared enlarged, measuring 56 × 32 × 43 mm and with a heterogeneous texture. The baby was breastfeeding well, with no reports of hematuria or vomiting—normal bowel movements. Since birth, the baby has experienced multiple hypertensive episodes and was placed on Isradipine 0.15 mg every 8 h when systolic blood pressure was over 105. BMP, electrolytes, and blood pressure were monitored until a radical nephrectomy was performed. BUN and creatine levels were within normal limits. Calcium levels were noted to be elevated at 10.7.

**Fig. 1:**
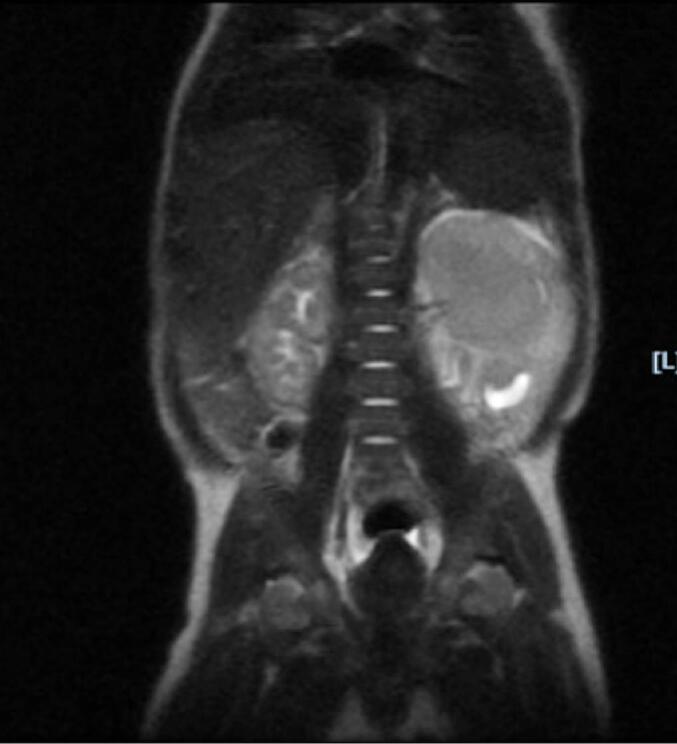
Coronal view revealing solid heterogeneously enhancing left renal mass measuring 5.3 x 5 cm.

**Fig. 2:**
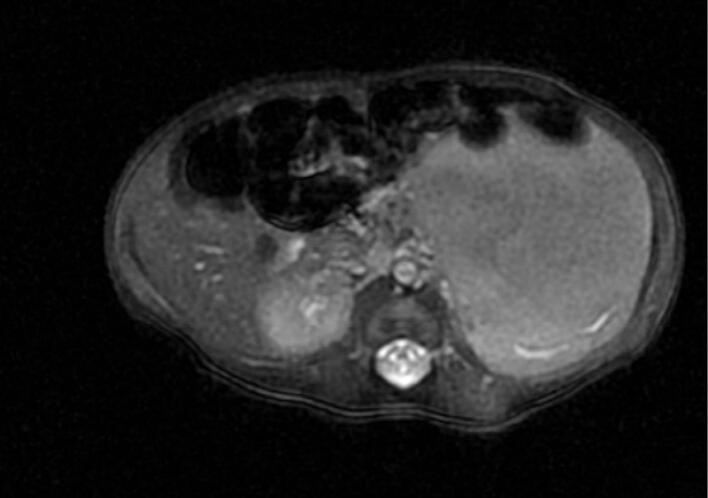
Axial view.

Ultrasound findings revealed that the left kidney measured 6.8 cm with a large heterogeneous solid mass infiltrating the hilum of the left kidney, demonstrating intrinsic flow and causing some dilatation of the renal collecting system. The tumor measures 5.8 × 3.6 × 5.3 cm. There is a normal-appearing adrenal gland superior to the left kidney. The MRI of the abdomen and pelvis w/ & w/o contrast in Fig. 1, Fig. 2 revealed a large heterogeneously enhancing mass centered in the upper to the mid pole of the left kidney measuring 5.3 × 5 cm. There is a sparing of the lower pole of the left kidney.

After a discussion with the patient's parents, a left radical nephrectomy was planned. The procedure was performed in the operating room under general anesthesia and lasted 68 min. Complete exposure to the lesion's anterior, lateral, and posterior surfaces was performed. The lesion was well-circumscribed without extending into the adjacent adrenal or retroperitoneal structures. The capsule appeared to be completely intact. The lesion was excised without any leakage or disruption of the tumor capsule. The lesion was mobilized upwards, and careful blunt cautery and sharp dissection on all lesion surfaces allowed it to be fully mobilized out of the retroperitoneum. The vascular pedicle was readily identified, as was the ureter. The artery and vein were carefully divided between clamps and tied off with #0 silk ties. The ureter was traced down to its entry into the bladder and excised, suture ligating the ureter at its entry into the bladder with 3-0 Vicryl suture.

The lesion was submitted to Pathology as a fresh specimen. After a discussion with the pathologist, the frozen section diagnosis was confirmed to be mesoblastic nephroma. Further attention to the retroperitoneum for the presence of grossly abnormal lymph nodes revealed none. There were very few lymph nodes in the paraaortic region. These were all normal. The retroperitoneum was irrigated copiously with warm irrigation fluid. There was no bleeding of the tumor bed. The incision was then closed in several layers.

Final pathology, seen in Fig. 4, Fig. 5, was consistent with congenital mesoblastic nephroma, classic type, although focal areas of hypercellularity were noted; the patient was diagnosed with stage I mesoblastic nephroma as it was utterly resected with an intact capsule and no renal vessel involvement. Therefore, no adjuvant chemotherapy was required for this patient. The patient will be followed-up with nephrology to monitor for the development of compensatory hypertrophy of the remaining kidney. In addition, the patient will undergo an ultrasound of the abdomen every three months for the first year of life and every six months during the second year of life.

## 3 Discussion

**Fig. 3:**
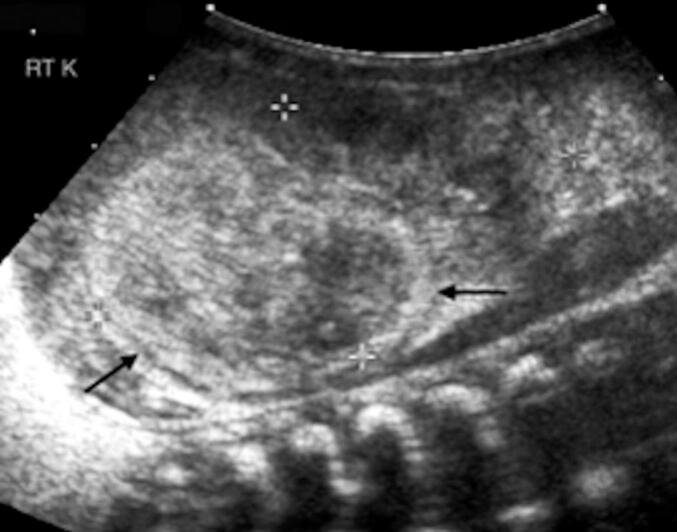
“Vascular ring” sign on ultrasound.

Once a neonate presents with an asymptomatic left upper quadrant abdominal mass, it is imperative to perform imaging to begin the differential diagnosis. Usually, an abdominal ultrasound is performed; CMN usually appears as a solid cyst with a homogenous appearance. Fig. 3 shows a common finding on ultrasound for CMN is described as a “vascular ring” [6]. Differentiating a benign CMN from a malignant nephroblastoma requires a radical nephrectomy. Image-guided biopsies should not be performed as they increase the risk of tumor spillage and local recurrence. Radical nephrectomy is currently the mainstay treatment for CMN. Follow-up ultrasounds every three months for the first year of life and then every six months for the second year are recommended to monitor for recurrence. If recurrence does occur, secondary resection is performed. In recent case studies, the chemotherapy trial for Wilm's tumor has been successfully used in patients with recurrence of CMN. This chemotherapy regimen consists of vincristine and actinomycin [7]. Further research in using Wilm's tumor for preventing recurrence should be evaluated in the future. In addition, monitoring calcium and renin levels may help monitor recurrence in the future.

**Fig. 4:**
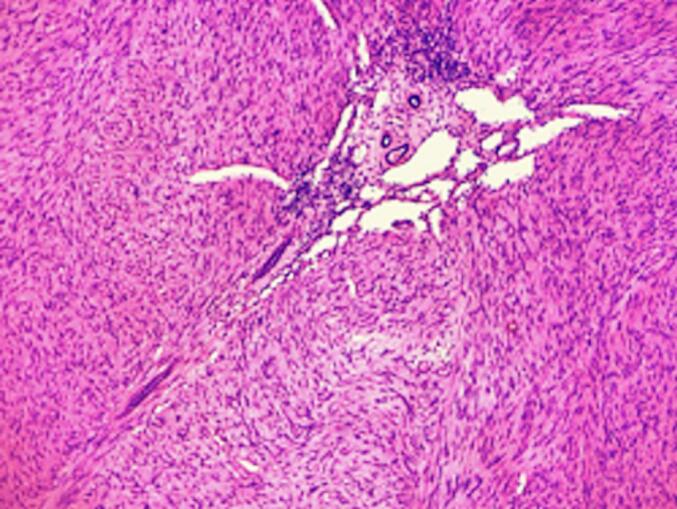
H&E stained left kidney. Spindle cells seen within normal kidney residue.

**Fig. 5:**
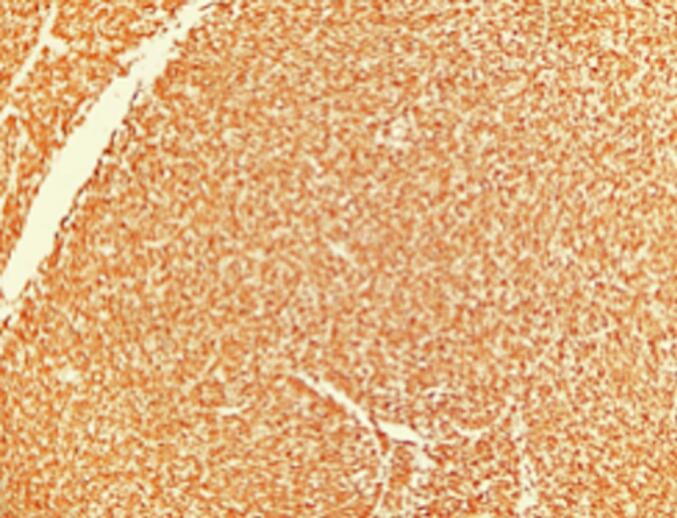
Left kidney, total nephrectomy. WT1 stain. Diffuse strong staining in the cytoplasm, as well as in some nuclei.

## 4 Conclusion

Identifying and diagnosing congenital mesoblastic nephroma should be performed in an expedited manner. Neonates with CMN are at risk for paraneoplastic syndromes such as hypercalcemia and secondary hypertension [5]. These patients also may have potential malignancy and metastases. Radical nephrectomy and pathological findings should be performed to determine the etiology of the renal mass to distinguish it from a nephroblastoma (Wilm's tumor), a malignant tumor.

## Patient consent

Written informed consent was obtained from the patient to publish this case report and accompanying images. A copy of the written permission is available for review by the Editor-in-Chief of this journal at request.

## Ethical approval

Not applicable.

## Funding

None.

## Guarantor

Kristen De Wilde.

## Research registration number

None.

## CRediT authorship contribution statement

Kristen De Wilde, MS: Study concept, data interpretation, writing the paper
Jamshed Zuberi, MD: Data interpretation, writing the paper.

## Conflicts of interest

None.
